# Frequency comb measurements for 6G terahertz nano/microphotonics and metamaterials

**DOI:** 10.1515/nanoph-2023-0869

**Published:** 2024-01-31

**Authors:** Guseon Kang, Younggeun Lee, Jaeyoon Kim, Dongwook Yang, Han Ku Nam, Shinhyung Kim, Soojeong Baek, Hyosang Yoon, Joohyung Lee, Teun-Teun Kim, Young-Jin Kim

**Affiliations:** Department of Mechanical Engineering, Korea Advanced Institute of Science and Technology (KAIST), Science Town, Daejeon 34141, South Korea; Department of Aerospace Engineering, Korea Advanced Institute of Science and Technology (KAIST), Science Town, Daejeon 34141, South Korea; Department of Mechanical System Design Engineering, Seoul National University of Science and Technology (SEOULTECH), Seoul 01811, South Korea; Department of Physics, University of Ulsan, Ulsan 44610, South Korea

**Keywords:** frequency comb, 6G, THz, topological photonics, metamaterials

## Abstract

Next-generation 6G communication holds the potential to revolutionize data transfer, enabling the realization of eXtended Reality (XR) with enhanced sensory experiences. To achieve this, advanced components such as high-performance intensity/phase modulators, waveguides, multiplexers, splitters, combiners, and filters operating in terahertz (THz) regime, specifically within the frequency range of 0.1–1 THz, are essential. However, existing microwave equipment and vector network analyzers designed for this frequency range suffer from limitations in resolution, stability, and accuracy when evaluating the intensity and phase responses of critical 6G THz devices. In this comprehensive review, we delve into the critical device requirements and emerging trends in next-generation 6G communication, essential performance evaluation parameters, comparisons between microwave and nano/microphotonic devices for testing, and the application of high-resolution THz sensors in 6G Internet-of-Things (IoT) scenarios. Notably, a frequency comb in the photonic regime emerges as the prime candidate for achieving precision evaluations of 6G networks and devices. Consequently, this review highlights the latest research in frequency comb measurements in the 6G THz frequency regime, with a particular emphasis on nano/microphotonic devices and metamaterials. The integration of frequency comb measurements into 6G and THz photonic devices and networks promises to accelerate the realization of high-density next-generation 6G communication.

## 1 Introduction

In the relentless pursuit of ever-evolving communication technologies, the emergence of eXtended Reality (XR) has ushered in a new era of immersive experiences, necessitating communication systems that can keep pace with the insatiable demands of this captivating realm. XR encompasses a spectrum of applications, including augmented reality (AR), virtual reality (VR), and mixed reality (MR), all of which require high-density communication systems to seamlessly blend digital content with the physical world [1], [2]. XR applications require high data rates, low latency, and robust connectivity to deliver immersive experiences. These applications encompass a wide range of scenarios, from interactive gaming and remote medical procedures to industrial training and architectural visualization. High-density communication in XR is characterized by the need to support a multitude of connected devices and users in close proximity while maintaining high-quality, synchronized content delivery. This necessitates advanced techniques for spatial multiplexing, interference mitigation, and efficient spectrum utilizations.

The transition from 5G to the anticipated 6G communication system is driven by the escalating demands of Extended Reality (XR) applications. The promise of 6G lies in its integration of high-frequency terahertz (THz) communication, necessitating the development of cutting-edge components like waveguides [3], [4], modulators [5], [6], [7], [8], filters [9], [10], [11], amplifiers [12], and detectors [13], [14], [15]. The validation and characterization of these components require the establishment of THz frequency standards for precise assessment of amplitude and phase characteristics essential for 6G communication. The evolution introduces THz frequencies (typically in the range of 100 GHz to 10 THz) to address the data rate requirements of XR and emerging applications. Overcoming challenges such as atmospheric absorption and limited propagation range is crucial for the viability of 6G THz communication, requiring innovations in antenna design [12], beamforming [16], [17], [18], and signal processing [16], [19].

The impending emergence of 6G wireless technology, operating in the THz frequency range from 100 GHz to 10 THz, signifies a revolutionary leap in communication networks [20]. Compared to its predecessor, 5G, 6G is poised to outperform on multiple fronts, boosting peak data rates from gigabits per second (Gb/s) to terabits per second (Tb/s). Reduced latency, from milliseconds in 5G to microseconds in 6G, opens avenues for latency-sensitive applications like driverless vehicles and remote surgery. 6G aims for ubiquitous connectivity through enhanced satellite and drone networks, even in rural regions, with AI integration optimizing network operations [21]. This transformative breakthrough envisions applications ranging from high-fidelity augmented reality to brain–computer interfacing, requiring the development of critical 6G THz devices and equipment, including THz transceivers [22], [23], metamaterials [5], [22], [23], [24], and quantum devices [25], [26], [27]. Advanced antenna technologies and dynamic spectrum sharing mechanisms will redefine connectivity, while AI integration will optimize resource allocation and network management.

In this paper, we embark on a journey through the fascinating landscape of 6G THz communication, elucidating the compelling amalgamation of technology and innovation required to drive XR to its zenith. We explore the high-density communication requirements inherent to XR and the inherent challenges posed by the integration of THz frequencies. Delving into the core of 6G communication, we dissect the multifaceted requirements of its integral components, highlighting the critical role they play in realizing the full potential of XR. Additionally, we delve into the vital importance of THz frequency standards, utilizing optical frequency comb to enhance precision, serving as a fundamental element for comprehensive testing and characterization. This contributes to the progress of reliable and efficient 6G THz communication systems. High-density communication, THz frequency integration, specialized components, and THz frequency standards collectively form the cornerstone of this technological evolution, promising to deliver immersive XR experiences and unlocking the full potential of 6G THz communication. As we traverse the evolving landscape of 6G THz communication, our endeavor is to elucidate the intricate interplay of these elements and their cumulative contribution to the realization of immersive XR experiences.

## 2 High-density THz communication requirements

**Figure 1:**
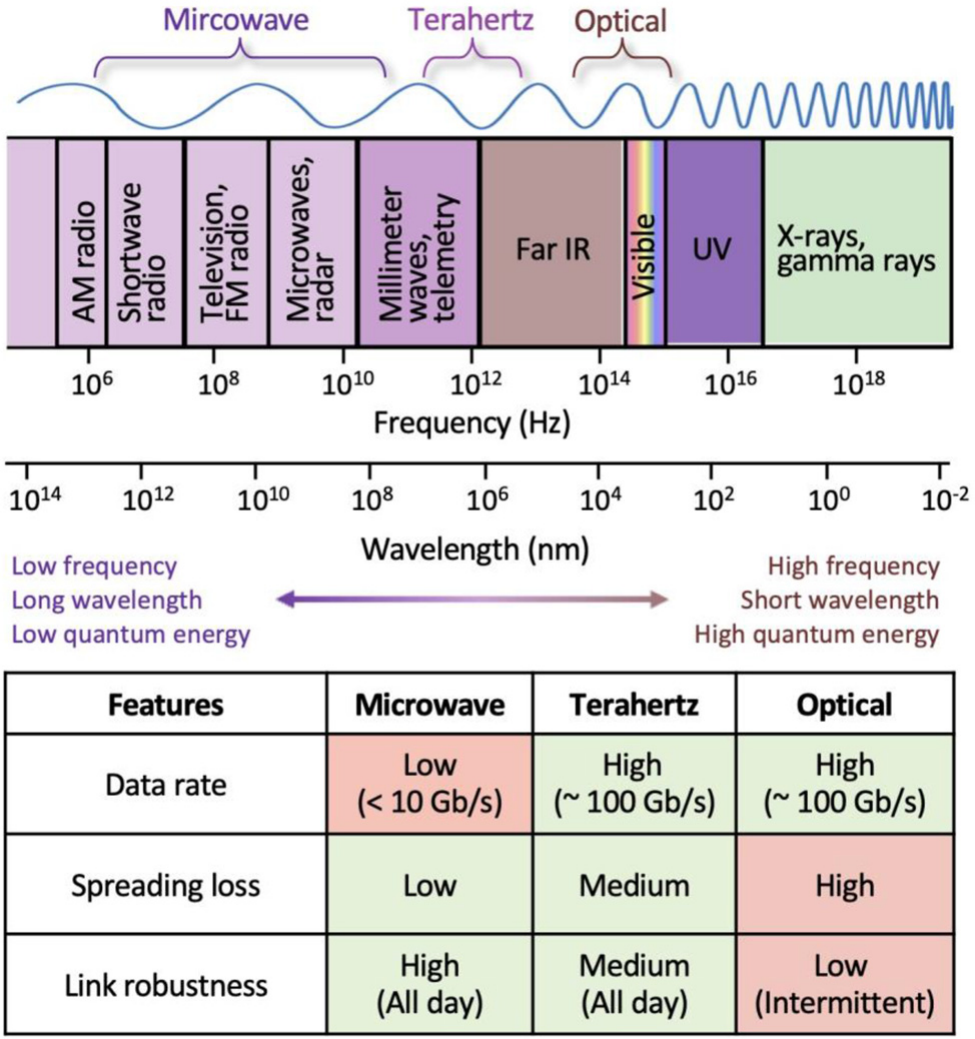
Comparison of hardware performance and features in wireless communication sources: microwave, terahertz, and optical frequencies in the electromagnetic spectrum.

Wireless communication resources can broadly be categorized into microwaves, THz, and optical frequencies (Figure 1). These resources manifest in the electromagnetic spectrum with distinct frequencies and wavelengths, resulting in different characteristics. Microwaves are represented by low frequencies and long wavelengths, possessing lower quantum energy. In contrast, optical frequencies exhibit relatively higher frequencies, shorter wavelengths, and higher quantum energy. THz and optical frequencies, being higher in carrier frequencies and offering broader useable bandwidth compared to traditional microwaves, have gained attention for their potential in enabling high-capacity data communication. As the demand for high-capacity wireless communication grows, the trend in the carrier frequencies of communication resources is shifting toward higher frequencies, suggesting the potential utilization of the THz band within the 0.1–10 THz range.

THz waves occupy an intermediate position between microwaves and optical frequencies. While THz waves share some characteristics with omnidirectional microwaves, they also exhibit the directional nature typical of optical frequencies. This unique property has inspired the design of THz components, drawing inspiration from optical devices [28], [29]. Spreading loss, inversely proportional to the square of the frequency [30], is more pronounced in THz and optical frequencies compared to microwaves. The atmospheric conditions, specifically the presence of water and oxygen molecules for THz frequencies and water and carbon molecules for optical frequencies, contribute to significant propagation losses [14]. Overcoming these challenges necessitates high-power transmitters or highly sensitive receivers. However, the development of THz transceivers lags behind other frequency bands, capable of generating power at levels approximately 2–3 orders of magnitude lower, with insufficient measurement sensitivity. In light of these challenges, the appropriate frequency selection becomes crucial, especially considering the absorption windows created by water and oxygen molecules in the atmosphere [28]. The 1–10 THz range, with numerous absorption lines, is being considered due to its higher carrier frequency compared to traditional microwaves. The sub-THz range, specifically within the frequency range of 0.1–1 THz, emerges as a viable option for potential applications.

THz regime possesses advantages over conventional communication wavelengths, making it a suitable candidate for implementing non-terrestrial networks [31], [32], [33]. Firstly, it allows for the construction of low-power efficient systems. THz, with its high carrier frequency and wide bandwidth, can achieve much higher data transmission speeds through simple forms of signal modulation, such as on-off keying (OOK) [28], [34], binary phase-shift keying (BPSK) [7], [35], and quadrature phase-shift keying (QPSK) [36], [37], [38]. This stands in contrast to traditional RF communication bands, such as S-band (1.7–2.7 GHz), Ku-band (10.6–15.7 GHz), and Ka-band (17.3–31 GHz), which rely on complex modulation/demodulation for optimal bandwidth utilization [31]. The use of the simplest modulation format enables the construction of low-power systems. Moreover, THz waves exhibit characteristics conducive to building compact systems. Since antenna gain is proportional to the square of the frequency, THz antennas can achieve higher gains with smaller sizes compared to RF antennas [39]. Furthermore, the potential for system miniaturization is expected even in comparison to FSO communication. A practical challenge with FSO link, considered a competitive technology to THz links, is the necessity for heavy mechanical beam alignment devices to precisely align the beams between transmitters and receivers. THz waves, with wavelengths that are 2–3 orders of magnitude longer than optical frequencies, demonstrate insensitivity to misalignments in position and angle [40]. The inherent property of THz waves, facilitating easy alignment of positions and angles, positions THz links as a promising technology for seamless, high-capacity communication.

**Figure 2:**
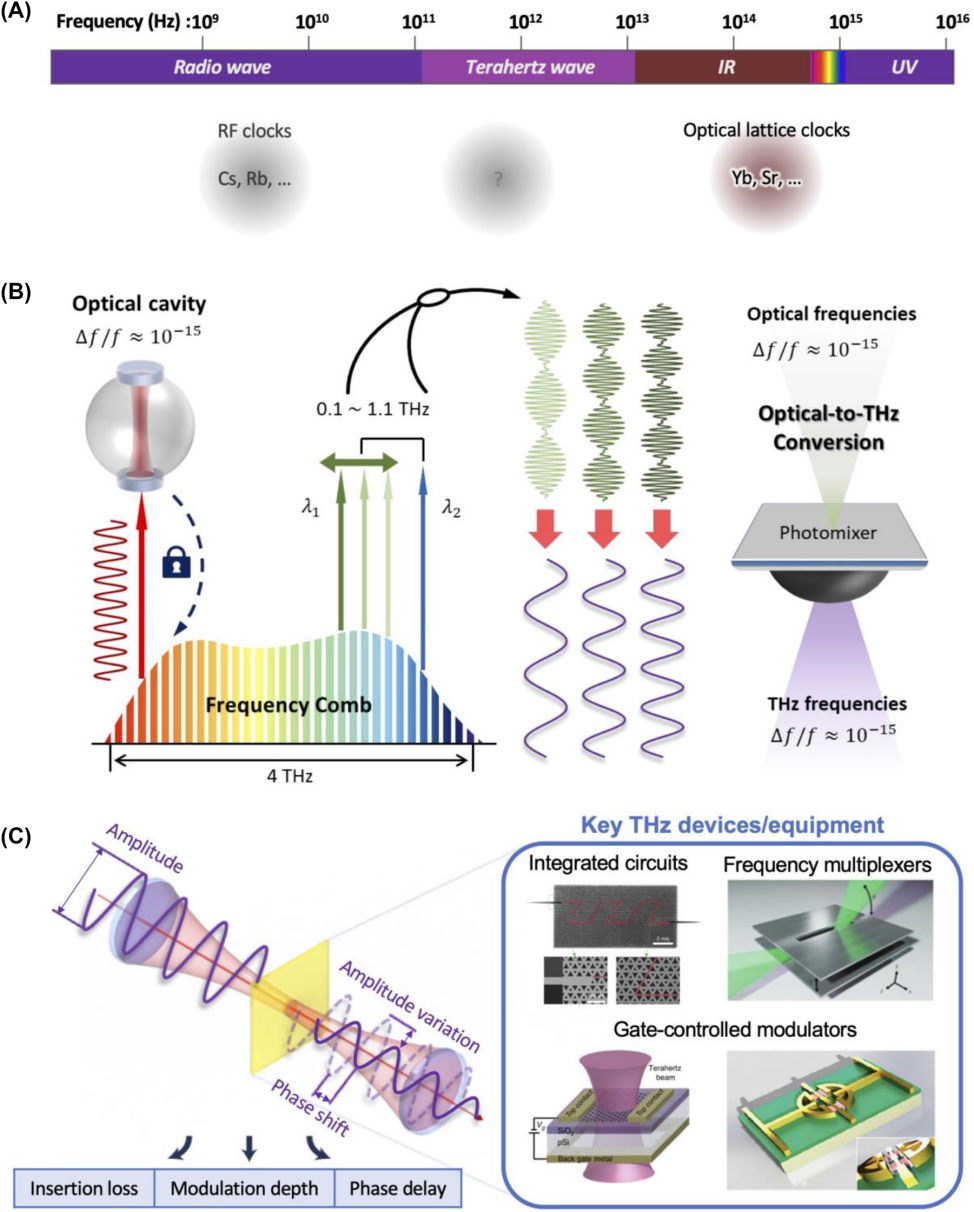
Requirements for XR realization through 6G THz communication. (A) Frequency standard for THz region to realize accurate characterization of 6G THz communication components. (B) Ultra-stable THz synthesis referenced to the optical clock, through the coherent optical-to-terahertz down-conversion [41]. (C) Comprehensive analysis of THz equipment and devices: examining spectral amplitude and phase variations in integrated circuits [42], frequency division multiplexers [43], and gate-controlled modulators [8], [44]. (C) Reproduced with permission [42]. Copyright 2020, Springer Nature. Reproduced with permission [43]. Copyright 2015, Springer Nature. Reproduced with permission [8]. Copyright 2012, Springer Nature. Reproduced with permission [44]. Copyright 2018, De Gruyter.

As 6G THz communication components become increasingly complex, it becomes imperative to establish THz frequency standards for accurate characterization. These standards are crucial for evaluating the amplitude and phase characteristics of components with precision, enabling the development of reliable and high-performance 6G THz communication systems. Calibration techniques and measurement methodologies in the THz domain will play a pivotal role in ensuring the quality and interoperability of 6G THz components (Figure 2). The relentless pursuit of THz communication in the context of 6G networks necessitates precise and reliable methodologies for testing the amplitude and phase characteristics of 6G communication components. As these components operate in the uncharted territory of THz frequencies, the establishment of THz frequency standards becomes paramount.

The stability of frequency is highly dependent on the chosen frequency standard (Figure 2A). Many research laboratories commonly use radio-frequency (RF) band clocks or frequency standards such as Rb and Cs [45]. There is a trend toward transitioning from RF clocks to optical clocks, driven by the advantage of optical frequencies having significantly higher stability compared to traditional radio frequencies [45]. The exceptional stability of optical clocks is being harnessed by utilizing the optical frequency comb to extend their precision to other frequency ranges. Over the past decade, research has successfully enhanced the stability of 10 GHz microwave frequencies to the level achieved by optical lattice clocks. However, when examining light sources in the THz range, the stability of optical frequency standards is not fully utilized. Research predominantly relies on RF clocks, referencing sources like Rb, Cs, and hydrogen masers, resulting in frequency stabilities at the 10^−12^ level [46], [47], [48]. To address this limitation, ongoing research aims to transfer the outstanding stability of optical clocks to the THz range [41]. The goal is to significantly elevate the stability of traditional THz light sources by incorporating the precision of optical clocks.

THz radiation faces challenges in efficient generation, creating the “Terahertz Gap” between 0.1 and 10 THz [49]. Electronic and photonic methods, though promising, struggle with compact, high-power, and room-temperature THz sources. Despite these challenges, a photonic approach using frequency down-conversion has shown promise, leading to the development of stable and accurate THz sources. Over the past two decades, advancements in optical frequency comb and technologies like THz quantum cascade lasers (QCLs) and direct optical heterodyning have contributed to stable THz wave generation, addressing some limitations posed by traditional methods. The emergence of stable and precise THz sources is closely tied to the development of the optical frequency comb as a photonic frequency divider [50], [51], [52]. THz QCLs represent a significant advancement in this context, being capable of direct phase-locking to a frequency comb [53], [54]. When QCLs are locked to a continuous wave (CW) frequency reference, they generate high-power THz waves with a broad tuning range exceeding 1 THz, spanning from 1 to 5 THz, although their continuous tuning range has to be narrowly restricted.

Alternatively, stable THz waves can be efficiently produced by directly optically heterodyning CW lasers through photomixers like uni-traveling-carrier photodiodes (UTC-PDs) [55], [56] or high-speed p-i-n photodiodes [57], [58], [59]. Using such photomixers, THz waves with suppressed phase noise can also be generated through optical frequency division, utilizing soliton microcombs stabilized at a high repetition rate [46], [60], [61]. Although microcomb-based sources exhibit comparatively better stabilities between 0.1 and 1.5 THz, challenges in regulating cavity length significantly limit their tuning potential. Leveraging the microwave H-maser, the best phase noise, and frequency stability levels achieved so far were −40 dBc/Hz at 1 Hz and 2 × 10^−13^ at 1 s, respectively [46]. Recent work has introduced a photonic strategy for THz synthesis through the photomixing of two coherently obtained comb lines from the source comb [41], as shown in Figure 2B. This approach brings stability to the optical frequency comb in the THz range, achieving an extremely low phase noise of −70 dBc/Hz at a 1-Hz offset. THz frequencies ranging from 0.10 to 1.10 THz are generated, and the reported frequency instability at 0.66 THz is 4.4 × 10^−15^ at 1-s integration. Notably, this recent work outperforms state-of-the-art counterparts by demonstrating superior performance at a 1-Hz offset, showcasing a remarkable 30 dB reduction in the noise floor. While QCLs injection-locked to a frequency comb [62] and stabilized microcombs [60] have achieved lower noise floors at offset frequencies above 100 Hz, the current noise floor of the synthesizer is limited by the photomixer’s insufficient strength for creating THz waves, a limitation expected to be addressed soon with the introduction of high-power photomixers.

THz frequency range faces challenges in the development of transceivers and passive components compared to other frequency domains [29]. This limitation hampers various applications, including THz wireless communication [28], [63], [64], [65], molecular spectroscopy [66], [67], and nondestructive testing (NDT) [68], [69], [70]. Consequently, ongoing research focuses on the fabrication of components such as waveguides [16], [19], [42], frequency-division multiplexers [71], [72], [73], and active modulators [5], [6], aiming to integrate them into practical applications. Despite the utilization of diverse components in application validation, there remains a significant gap in verifying the frequency and phase changes that occur in THz waves after passing through these components. This gap arises from limitations in the performance of systems used to validate component characteristics. The integration of stable sources based on frequency standards is anticipated to enable a thorough examination of the size and phase-related spectral variations introduced by THz components (Figure 2C).

THz frequency standards must exhibit exceptional frequency stability and spectral purity. The THz spectrum is inherently congested with interference sources, and precise measurements require standards that can produce highly stable and pure THz signals. Advanced techniques such as optical frequency comb and cryogenic oscillators are being explored to achieve the necessary level of frequency precision in the THz regime [41], [46], [60], [61]. The calibration accuracy of THz frequency standards is pivotal for ensuring the reliability of amplitude and phase measurements of 6G components. Calibration techniques that account for environmental factors, signal loss, and component imperfections must be developed to achieve accurate and traceable measurements. This involves the establishment of reference standards and calibration protocols specifically tailored for THz frequencies. 6G communication components span a wide range of frequencies within the THz spectrum. Consequently, THz frequency standards must be capable of operating across this broad bandwidth to accommodate the diverse characteristics of 6G devices. The ability to generate THz signals with high fidelity over a wide frequency range is essential for comprehensive component testing. Low phase noise is critical for characterizing the phase characteristics of 6G components, especially in high data-rate applications. THz frequency standards must exhibit minimal phase noise, which is challenging at these frequencies due to technical limitations. Innovative solutions, such as cryogenic cooling and ultra-stable resonators, are being investigated to minimize phase noise in THz sources. In conclusion, THz frequency standards represent a critical technological foundation for the evaluation of amplitude and phase characteristics in 6G communication components (Figure 2C). Their development and deployment require a concerted effort to address the unique challenges posed by the THz frequency regime, including frequency stability, calibration accuracy, broadband operation, low phase noise, portability, and compatibility. The realization of reliable THz frequency standards is pivotal in enabling the successful deployment of 6G communication systems and unlocking their transformative potential in the era of high-frequency, high-data-rate wireless networks.

## 3 Critical 6G THz devices, equipment, and networks

The forthcoming era of 6G communication is poised to unleash unprecedented levels of connectivity and data throughput, revolutionizing how we interact with and utilize wireless networks. Key to the realization of 6G’s promises is the intricate and highly specialized components that form its foundation. In this section, we provide a concise overview of the technological requirements for essential 6G communication components, including waveguides, modulators, filters, amplifiers, and detectors. Waveguides in 6G communication systems must exhibit exceptional efficiency in guiding high-frequency electromagnetic signals, particularly in the THz frequency range. These waveguides should minimize signal loss and dispersion while supporting multi-mode operation to accommodate diverse communication needs [4], [42]. Innovative materials, such as advanced metamaterials and low-loss dielectrics, are crucial to achieving these goals [4], [42]. Miniaturization and integration capabilities are equally vital to ensure compatibility with compact and high-density 6G device designs [16], [19], [42]. Modulators are essential for manipulating and encoding data onto the THz carrier signals in 6G systems. To meet 6G’s ambitious data rate requirements, modulators must operate seamlessly in the THz frequency range while offering high modulation depth and ultra-fast response times [5]. Advanced electro-optic or electro-absorption modulators, integrated with semiconductor [74], [75], [76] or plasmonic structures [77], [78], are being explored to enable efficient signal modulation at these extreme frequencies. Filters play a pivotal role in 6G communication by facilitating spectral shaping, interference mitigation, and channel selection. In the THz regime, filters must be designed to be compact, low-loss, and highly selective to accommodate the dense spectrum of 6G signals [9], [10], [11]. Novel filter technologies, including metamaterial-based designs [79], [80], [81] and reconfigurable filter banks, are being investigated to address these demanding requirements. Amplifiers are indispensable components for boosting signal strength in 6G communication systems, especially in the presence of significant path losses at THz frequencies [12]. Power-efficient and low-noise amplifiers capable of operating at THz frequencies are imperative for sustaining reliable and high-performance 6G connections. The development of solid-state and quantum amplifiers holds promise for meeting the stringent demands of 6G networks. Detectors in 6G communication systems must accurately receive and process THz signals, offering high sensitivity and low noise. State-of-the-art THz detectors, such as quantum-cascade detectors and bolometers, are being explored for their potential to deliver the required performance [28], [29]. Moreover, detector arrays and integration techniques are vital to accommodate the massive MIMO (Multiple-Input, Multiple-Output) systems envisaged for 6G [82].

**Figure 3:**
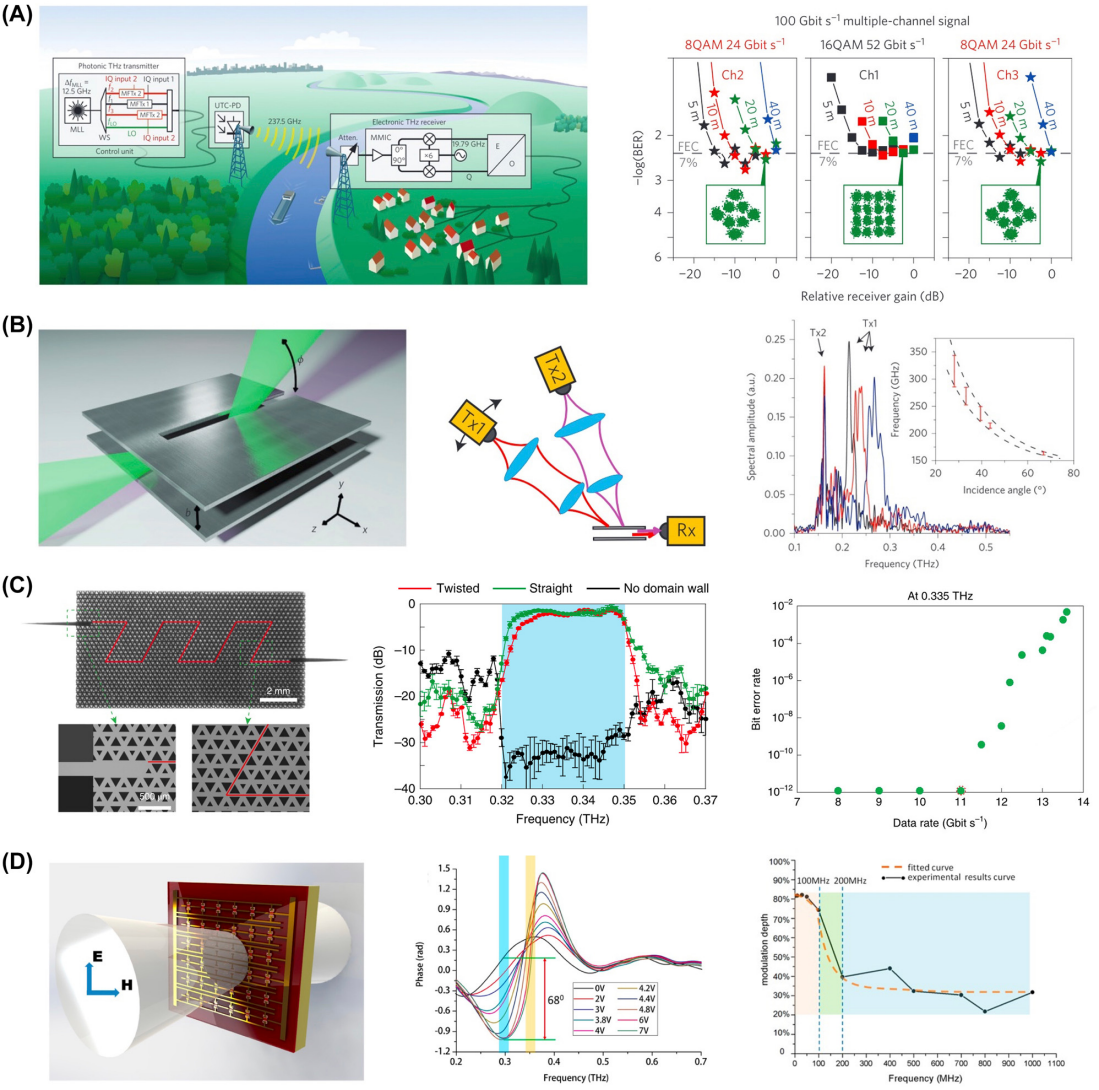
Crucial 6G terahertz (THz) devices essential for the implementation of THz 6G communication. (A) Photonically generated THz signals using an optical frequency comb, which provides comb modes for data modulation carriers f_1_,f_2_, f_3_, and unmodulated local oscillator f_LO_ [36]. (B) Frequency division multiplexer/demultiplexer for enhancing data capacity across a broad frequency range [43]. (C) THz integrated circuits enabling seamless integration of 6G THz communication while reducing size, weight, and power consumption [42]. (D) Gate-controlled THz modulator with the structure of a composite metamaterial that provides a basis for the development of effective and ultrafast dynamic devices for THz wireless communication [6]. (A) Reproduced with permission [36]. Copyright 2013, Springer Nature. (B) Reproduced with permission [43]. Copyright 2015, Springer Nature. (C) Reproduced with permission [42]. Copyright 2020, Springer Nature. (D) Reproduced with permission [6]. Copyright 2015, American Chemical Society.

Optical frequency comb-based systems, with their stable and narrow comb lines, have improved spectral efficiency in wavelength-division multiplexing (WDM) by allowing smaller guard bands, traditionally several GHz wide, to reduce interchannel crosstalk [83], [84], [85]. This advancement has led to data transmission rates of several hundred Tb/s, underscoring the effectiveness of WDM and the role of coherent comb lines in enhancing digital signal processing [85]. The extension of this stability to the THz regime could further enhance THz communications. Utilizing the optical frequency comb at 1550 nm enables accessing bandwidths over 4 THz, with the potential to reach up to 10 THz, thereby significantly boosting data transmission rates. Developing robust sources for broader THz spectra and chip-scale system integration is essential for advancing efficient [86], stable multichannel communication in the THz range. A recent study introduces a single-input and single-output (SISO) wireless communication system at 237.5 GHz, achieving a groundbreaking data rate of 100 Gb/s over a 20-m distance [36], as illustrated in Figure 3A. The breakthrough results from combining THz photonics and electronics, generating a narrow-band THz carrier through the photonics–electronics hybrid system. The research further presents a SISO wireless link operating at 237.5 GHz, achieving a data rate of 100 Gb/s without resorting to MIMO technology. Previous wireless links at lower frequencies required MIMO setups for comparable data rates. The study demonstrates the potential of combining THz photonics and electronics to pave the way for high-capacity wireless communications in the THz frequency range. The integration of optical polarization multiplexing and MIMO technology, coupled with advancements in photonic and electronic subcomponents, holds the potential to significantly enhance the system’s performance, allowing for data rates of up to 200 Gb/s. Envisaging the scaling toward a Tb/s system, this advancement leverages optical techniques such as wavelength division multiplexing, frequency division multiplexing, and spatial multiplexing. Furthermore, incorporating millimeter-wave monolithic-integrated circuits (MMICs) [87], [88] and high-gain Cassegrain antennas (>50 dBi) at both the transmitter (Tx) and receiver (Rx) sides could extend the capabilities to enable long-distance communication exceeding 1 km. This holistic approach not only enhances data transfer rates but also opens up possibilities for reliable and compact communication systems with broad applications in high-speed and long-range wireless communication.

To achieve the targeted data rate of Tb/s, multiplexing and demultiplexing functionalities for independent data streams in signal channels are crucial in high-data-rate communication. Networks are actively researching ways to enhance data processing capacity by increasing the number of distinguishable noninterfering subcarrier frequencies, a strategy widely utilized in fiber optic networks and microwave wireless communication. In the THz band, there has been a lack of existing frequency division multiplexing components for such roles. A recent study introduced a leaky-waveguide, a device leveraging the directional characteristics of THz wireless signals to perform multiplexing and demultiplexing between free space and a waveguide across a broad frequency range [43], as shown in Figure 3B. This device, based on a proven metal parallel-plate waveguide designed for manipulating THz signals, includes a narrow slot in one of the metal plates, allowing a portion of the guided wave to leak into free space for demultiplexing. The frequency of radiated radiation at a given angle is determined by phase-matching constraints, resulting in guided waves being radiated into free space at different angles depending on the frequency. For multiplexing, the structure facilitates frequency division multiplexing and demultiplexing by enabling incoming waves from free space to couple into the waveguide when meeting phase-matching conditions. The demonstrated component showcased effective frequency division multiplexing and demultiplexing over a 1-octave bandwidth, with potential extension to a broader THz range, independent of the transparency of dielectric materials. It offers control over the frequency and bandwidth of multiple channels by adjusting the spacing and shape of the waveguide.

Communication demonstrations using this component achieved a speed of 50 Gb/s with QPSK-modulated signals in the 220–330 GHz frequency range [71]. Additionally, a novel approach for locating a mobile receiver in a highly directional network was demonstrated [72]. This approach avoids the sequential trial-and-error method and enables precise location determination in a single shot using broadband sources and receivers. The versatility of this platform may lead to future developments, including the advancement of modulators with high degrees of freedom. This could involve innovations such as controlling leakage rates through variations in slot width based on length, allowing for relative adjustments in multiplexing efficiency and compensation for spatial profiles of different frequency channels [43]. Additionally, there is potential for an active device implementation by manipulating the shape of the waveguide using electromagnetic components.

To fully exploit the potential of THz waves, it is crucial to develop integrated circuits hosting hybrid electro-optical components on a complementary metal–oxide–semiconductor (CMOS) compatible platform [17]. THz integrated circuits play a vital role in improving the performance and reliability of THz functional components while reducing size, weight, and power consumption. This advancement is pivotal in the era of high-speed on-chip communication, enabling the seamless integration of new communication devices and facilitating the processing and computation of massive amounts of data for 6G and beyond wireless networks [16], [19]. The development of THz integrated circuits also promises significant enhancements across various fields, including biological diagnostics [89], [90], high-resolution imaging [91], food and pharmaceutical quality inspection [92], and nondestructive testing [68], [69], [70]. Although challenges such as the lack of compact THz sources and detectors have hindered THz integrated circuit development, recent progress in miniaturized sources based on electronics and photonics opens avenues for designing low-cost, high-efficiency THz integrated circuits on silicon platforms.

Nevertheless, on-chip THz devices currently encounter obstacles, notably reflection and scattering losses, particularly at sharp bends or defects [93], [94], [95], [96]. The recently discovered topological phases of light confer distinctive attributes to photonic devices, enabling reflection-free propagation and resilience against impurities or defects [42], as illustrated in Figure 3C. The study compares measured results in valley Hall photonic crystal (VPCs) with straight or no domain walls, revealing a distinct dip in transmission between 0.32 THz and 0.35 THz in VPCs without domain walls, indicating a bulk bandgap. In contrast, VPCs with twisted or straight domain walls exhibit near-unity transmission within the bandgap, with an estimated minimum bending loss of <0.1 dB per bend, surpassing conventional THz photonic-crystal waveguides [96]. However, outside the bandgap, transmission becomes complex due to reflection and refraction. The research suggests topological kink states as excellent information carriers for on-chip THz communication, demonstrated in a communication experiment achieving error-free transmission at 11 Gb/s with a carrier frequency of 0.335 THz. These attributes are paramount for the efficacy of THz integrated devices. Leveraging the resilience of topological edge states alongside a low-loss silicon platform holds immense potential for markedly improving the performance of THz devices. Key advancements in the field of chip-scale topological photonics include the development of versatile and efficient waveguides [97]. These include a 327 Gb/s multicarrier broadband waveguide interconnect [98], a 100 Gb/s topological antenna designed for wireless communication between chips [99], and an arbitrarily shaped waveguide capable of functioning as a four-port splitter and router [100]. Additionally, there have been significant strides in topological active devices, such as an electrically adjustable notch filter with over 20 dB suppression depth [101], and a phototunable 160 Gb/s waveguide with demultiplexing capabilities [102]. These innovations are set to revolutionize the realm of terahertz integrated circuits and high-speed interconnects.

The increasing demand for THz communication and imaging data transmission has prompted the need for advanced modulation techniques. Current active THz devices face limitations in meeting system demands. A recent study presents a novel composite metamaterial structure combining an equivalent collective dipolar array with a double-channel heterostructure [6] to create an effective, ultrafast, and all-electronic grid-controlled THz modulator (Figure 3D). The modulator achieves a groundbreaking 1 GHz modulation speed and an 85 % modulation depth during real-time dynamic tests, along with a 1.19 rad phase shift. Tested in a wireless free-space modulation THz communication system, the modulator exhibits 0.2 Gb/s eye patterns, providing a foundation for the development of efficient and ultrafast dynamic devices for THz wireless communication and imaging systems. Experimental results indicate that the modulator’s resonant frequency, initially at approximately 0.27 THz, blueshifts to 0.351 THz with increasing applied voltage, depleting the two-dimensional electron gas (2DEG) in the double-channel heterostructure. This voltage-induced resonant mode conversion leads to significant amplitude modulation, achieving an 85 % modulation depth at 0.351 THz. The gate voltage also influences the equivalent dielectric permittivity, resulting in a THz wave phase shift. The modulator demonstrates stable sinusoidal waveforms up to 1 GHz modulation speed, allowing for loading up to 1 Gb/s data on the carrier wave. However, impedance mismatch and packaging issues reduce modulation depth, especially above 200 MHz.

## 4 6G THz metrology and standard requirements: resolution, stability, accuracy, range, and speed

THz spectroscopy stands as a potent tool for delving into a diverse array of physical phenomena, encompassing rotational and vibrational transitions in molecules, low-energy excitations, and carrier dynamics in electronic materials [103]. The interaction strength of THz radiation, characterized by millielectronvolt photon energies, is particularly pronounced in systems with lifetimes in the picosecond range and energy transitions within the millielectronvolt range. This broad category includes entities like bound electrical charges, free charge plasmas, tightly confined charge plasmas, excitons, transient molecular dipoles, phonons in crystalline solids, and weakly bonded molecular crystals. Beyond fundamental research, THz spectroscopy finds valuable applications in the analysis of THz devices, such as low-loss waveguides, broadband absorbers, and active modulators. However, the characterization of these devices traditionally relies on classic THz spectroscopic methods, specifically THz time domain spectroscopy (THz-TDS). Despite its widespread use, THz-TDS has inherent limitations in terms of resolution, precision, and reliability. The future progress of THz applications critically depends on the development of high-efficiency interconnecting technology that minimizes signal loss across various equipment and components. Therefore, it is imperative to meticulously evaluate the frequency and phase characteristics of these components. In this section, we delve into the operational principles and performance requirements of commonly utilized tools for component characterization, with a focus on RF-based vector network analyzers (VNAs) and THz-TDS. Additionally, we introduce THz precision spectrometers designed to meet the demanding requirements of advanced THz applications.

THz vector network analyzer (VNA) [108], [109], [110] functions as an essential RF-based measurement device, enabling precise measurements of complex electromagnetic signals. While conventional VNA equipment is commonly utilized for measurements in the RF frequency range, for measurements at the high frequencies of the THz range, a distributed architecture is typically employed. This involves using a stimulus signal that is frequency multiplied or up-converted to elevate it to the frequency range in which the sample or device under test (DUT) operates. Additionally, a frequency extender is utilized to down-convert the response signals generated by the sample. Its capability to discern reflection, transmission, and key properties of the DUT makes it a vital resource for understanding intricate electromagnetic behaviors within the THz range. However, this method still has limitations, as it can only measure a restricted frequency range in the order of several hundred GHz. The measurement range typically includes 0.11–0.17 THz to 1.1–1.7 THz. This stands in contrast to optical-based THz time-domain spectroscopy (THz-TDS) [104], [105], [106], [107], [111] and CW THz spectroscopy [58], [68], which can measure a broader range of 2.5–6 THz.

**Figure 4:**
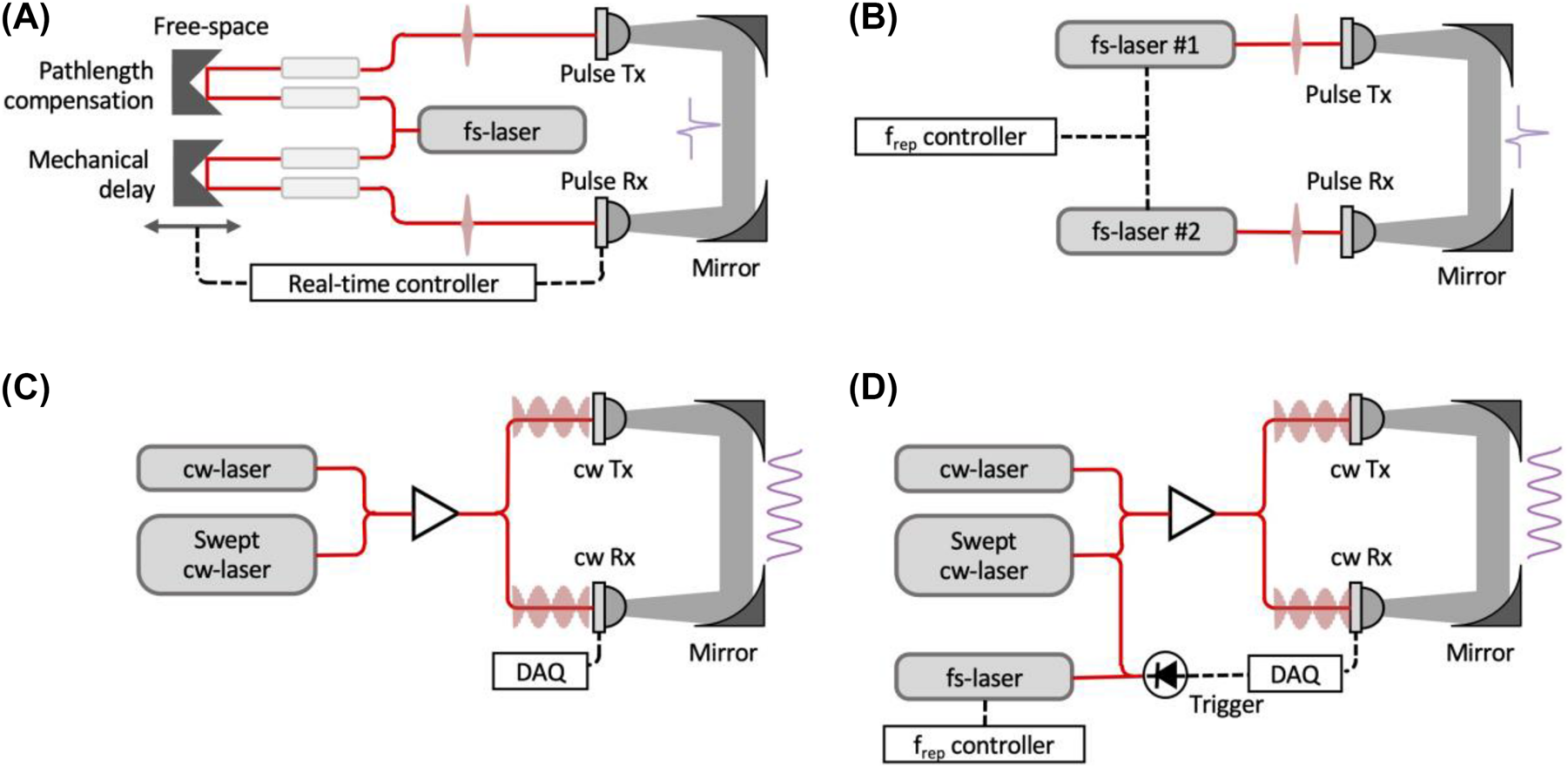
Configurations of terahertz spectrometers for assessing the performance of THz components. (A) Terahertz time-domain spectroscopy (THz-TDS) with a mechanical scanning of THz pulse in the time domain [104]. (B) Asynchronous optical sampling THz time-domain spectroscopy (ASOPS-THz-TDS) consisting of two mode-locked femtosecond lasers with slightly mismatched repetition rates [105], [106], [107]. (C) Continuous-wave (CW) THz spectroscopy via a photomixing of a near-infrared (NIR) CW laser and a continuously tunable NIR CW laser [68], [70]. (D) Frequency-comb-referenced CW THz spectroscopy with frequency-comb calibration.

THz-TDS has emerged as a dependable method known for its remarkable bandwidth and enhanced temporal resolution [112]. The utilization of coherent detection in the time domain, particularly for THz pulses lasting less than 1 ps, allows for the determination not only of the pulse’s intensity but also of the transient electric field. The Fourier transformation of this transient electric field facilitates the direct measurement of each spectral component, encompassing both amplitude and phase. These amplitude and phase measurements play a crucial role in determining a sample’s refractive index and absorption coefficients. In a typical THz-TDS system [104], as depicted in Figure 4A, a femtosecond laser is divided into two beam pathways. The first pulse train is directed toward the THz emitter, usually a photoconductive antenna, which generates THz radiation. These THz pulses interact with the sample before reaching the receiver, where they overlap with the second laser pulse train. The second laser pulse train is temporally adjusted using a mechanical delay stage to synchronize with the arrival of THz pulses. However, the operational speed of the mechanical delay line is limited by the substantial mass of its components. As a result, systems relying on mechanical delay lines can sample THz waves at a relatively slow pace. For instance, a typical commercial system is capable of scanning a delay window spanning 100 ps at a rate of 50 Hz.

To overcome the constraints associated with the measurement speed of existing systems, alternative techniques such as electrically controlled optical sampling (ECOPS) [111] and asynchronous optical sampling (ASOPS) [105], [106], utilizing two femtosecond lasers, have been proposed. The ECOPS system employs two femtosecond lasers, one with a fixed repetition rate and the other with a tunable repetition rate modulated relative to the first laser. This approach enables high measurement rates and dynamic ranges comparable to those achieved with mechanical delay. The time required to obtain a complete THz trace is determined by the difference in the two repetition rates, allowing for a measurement rate that can reach several thousand hertz with a sufficiently large difference. On the other hand, ASOPS utilizes two femtosecond lasers with slightly different repetition rates, and the repetition rate of the two femtosecond lasers is referenced to the frequency standard, as depicted in Figure 4B. Compared to methods involving a mechanical delay stage or ECOPS, ASOPS offers traceability to SI time and frequency standards with improved uncertainty and frequency accuracy. However, ASOPS has certain limitations. Femtosecond fiber lasers operating at a wavelength of 1550 nm have repetition rates ranging from tens of MHz to GHz, resulting in a broad scan range of 1–100 ns, which consumes significant measurement time. Additionally, the high measurement rate necessitates extremely wide bandwidth transimpedance amplifiers (TIA) and digitizers, impacting the signal-to-noise ratio as well as the system’s size and cost.

Figure 4C exhibits the setup of THz continuous-wave spectroscopy featuring a continuously tunable laser, enabling swift scanning across a broad THz spectrum up to 4 THz [68]. Recent advancements highlight continuous-wave spectrometers achieving a maximum measurement speed of 200 Hz, or through data averaging, satisfying a 4 THz bandwidth with a peak dynamic range of 117 dB [68]. This represents a notable innovation as these continuous-wave spectrometers match the performance of widely used THz-TDS systems but operate without femtosecond lasers and mechanical delay lines. Additionally, the adoption of cost-effective semiconductor chip-based continuous-wave lasers reduces overall expenses. However, despite this innovation, maintaining the frequency accuracy of THz waves remains a challenge, as it heavily depends on the initial calibration of lasers and is susceptible to fluctuations in ambient temperature and equipment aging. Nevertheless, ongoing efforts are dedicated to enhancing the stability and precision of continuous-wave THz technology, aiming to overcome these obstacles and solidify its position as a robust and reliable alternative to traditional THz-TDS.

Given the current inadequacy in the development of transceivers and active/passive devices in the THz band, precise frequency and phase measurement encounter limitations, primarily due to the absence of a dedicated and high-precision wavelength meter. The configuration depicted in Figure 4D capitalizes on advanced optical comb technology to ensure precise frequency definition by combining a THz band precision wavelength meter and a CW THz spectroscopy. This integration aims to establish the absolute THz frequency and enable accurate frequency and phase measurements in the THz band. The proposed system will incorporate a continuously tunable laser for wide-frequency-range scanning, accompanied by a frequency-fixed laser referenced to an optical frequency comb to ensure stable frequency output [113], [114], [115], [116], [117]. Despite the inherent frequency instability of the continuously tunable laser, the system will utilize frequency comb calibration to precisely define its absolute frequency [118], [119], [120]. By integrating this system into a THz generator and subsequently measuring the output with the combined setup, this approach will enable the precise measurement of a THz spectrum with a well-defined absolute frequency.

Table 1 provides a comparison of the spectroscopic performance of photonic-based THz spectrometers. The comparison includes parameters such as frequency bandwidth, bandwidth selectivity, frequency resolution, frequency accuracy and traceability to SI standard, and peak dynamic range. The observable bandwidth improves with increased measurement time or the number of averaging cycles, a trend observed in both THz-TDS and CW THz spectroscopy. THz-TDS typically exhibits a broad bandwidth, around 4 THz, while CW THz spectroscopy has been reported with bandwidths ranging from 1.1 THz [41] to a maximum of 4 THz [68]. The development of efficient THz continuous-wave generation technologies, such as improved photomixers or UTC-PDs, is expected to contribute to further enhancements in bandwidth.

**Table 1:** Spectroscopic performance of photonic-based THz spectrometers.

Methods	Frequency bandwidth	Bandwidth selectivity	Frequency resolution	Frequency accuracy	Traceability to standard	^a^Dynamic range	Reference
THz-TDS	4.5 THz	No	5 GHz	–	No	90 dB	Vieweg et al. [104]
ECOPS-THz-TDS	4.8 THz	No	1.4 GHz	–	No	68 dB	Yahyapour et al. [111]
ASOPS-THz-TDS	3 THz	No	50.5 MHz	6.2 × 10^−6^	Yes	43 dB	Yasui et al. [105]
	6 THz	No	1 GHz	5.7 × 10^−5^	Yes	39 dB	Klatt et al. [106]
	2 THz	No	2.5 MHz	8.4 × 10^−7^	Yes	40 dB	Hsieh et al. [107]
CW THz spectroscopy	2.75 THz	Yes	40 MHz	–	No	110 dB	Deninger et al. [58]
	4 THz	Yes	1 GHz	–	No	90 dB	Liebermeister et al. [68]
	1.1 THz	Yes	100 MHz	4.4 × 10^−15^	Yes	–	Shin et al. [41]

^a^For comparison, the peak dynamic range was scaled by assuming an averaging number of 1000.

THz-TDS, which samples THz pulses in the time domain and performs Fourier transformation, inherently lacks selectivity in observing specific frequency ranges. On the other hand, CW THz spectroscopy can have bandwidth selectivity in the frequency domain. While recent research emphasizes the capability for broadband measurements, leveraging bandwidth selectivity based on accurate frequency determination can lead to improved measurement speed or enhanced dynamic range. In the realm of frequency resolution, THz-TDS typically functions at the GHz level, whereas CW THz spectroscopy operates at the MHz level. Mechanically scanning-based THz-TDS systems face constraints imposed by the scanning step size, whereas ECOPS- and ASOPS-THz-TDS rely on the mode spacing or repetition rate of the femtosecond laser to determine resolution [105], [106]. Recent research has combined dual-comb spectroscopy with femtosecond laser repetition rate sweeping, achieving a notable frequency resolution of 2.5 MHz [107] (equivalent to the comb mode linewidth) rather than the mode spacing.

Advancements in frequency accuracy and traceability to frequency standards have been notable in both THz-TDS and CW THz spectroscopy. The accuracy of frequency is contingent on the employed frequency standard, often involving the stabilization of an infrared laser to a microwave frequency standard [46], subsequently converted to THz waves for precise assessment, with proposed stability levels below 10^−12^. A recent study has demonstrated the synthesis of ultra-stable THz waves with exceptional frequency stability at the 10^−15^ level, referencing an optical frequency standard [41]. A demonstration involving frequency scanning at 100 MHz intervals highlighted the potential for ultra-precise THz spectroscopy. Furthermore, the integration of a continuously tunable laser and frequency comb calibration, as depicted in Figure 4D, is anticipated to augment frequency stability, enabling enhanced frequency resolution in the sub-MHz range through the interpolation of instantaneous frequency of continuously tunable laser using frequency comb calibration [120].

Particularly in the case of dynamic range, the comparison assumes an averaging of 1000 times, and the scaled value is displayed as the peak dynamic range. In typical scenarios dominated by random noise, increasing the number of averages reduces random noise, thereby enhancing the dynamic range [68]. This implies that when the number of averages is increased tenfold, the dynamic range improves by 10 dB. The ASOPS THz-TDS can achieve precise spectroscopy by simultaneously using all the modes that compose the optical comb for frequency referencing [121], [122]. However, due to the limited optical power of each comb mode in the order of sub-nanowatts, increasing the number of averages is essential for achieving a high dynamic range. Yet, the dynamic range of this scheme is limited, reaching a level of 40 dB for 1000 averages [105], [106], [107], compared to the 90 dB dynamic range of conventional mechanically scanned systems [104]. Peak dynamic range refers to the largest dynamic range observed in the measured spectrum, typically occurring at 0.5 THz in THz-TDS and 0.1 THz in CW THz spectroscopy. As the range tends to increase gradually in the 0.1–0.5 THz range in THz-TDS, there might be constraints in evaluating components operating in the sub-0.5 THz range. The application of CW THz spectroscopy is promising, especially considering that candidate bands for THz communication fall within the low-frequency range of 0.1–0.5 THz.

## 5 Coherent transfer of photonic frequency comb to 6G THz signal sources

Although ultra-stable THz signals are essential in 6G wireless communication, the generation of high-performance THz waves with reference to international time standards requires sophisticated stabilization with a large system volume. Therefore, the phase-coherent distribution of the ultra-stable THz signals to distant places is requested for constituting a 6G communication network. The THz signals can be transferred through fiber networks or atmospheric free-space optical links in the form of THz waves or two optical signals that can be frequency-down converted by mutual heterodyning. The fiber network is stable but requires the pre-installed fiber network platform, whereas the free-space network can be actively constituted to arbitrary target locations, including satellites, high-altitude platform stations (HAPS), and drones. In this section, we elucidate the technological prerequisites for THz frequency standards, which are essential for the accurate characterization and validation of 6G communication components.

**Figure 5:**
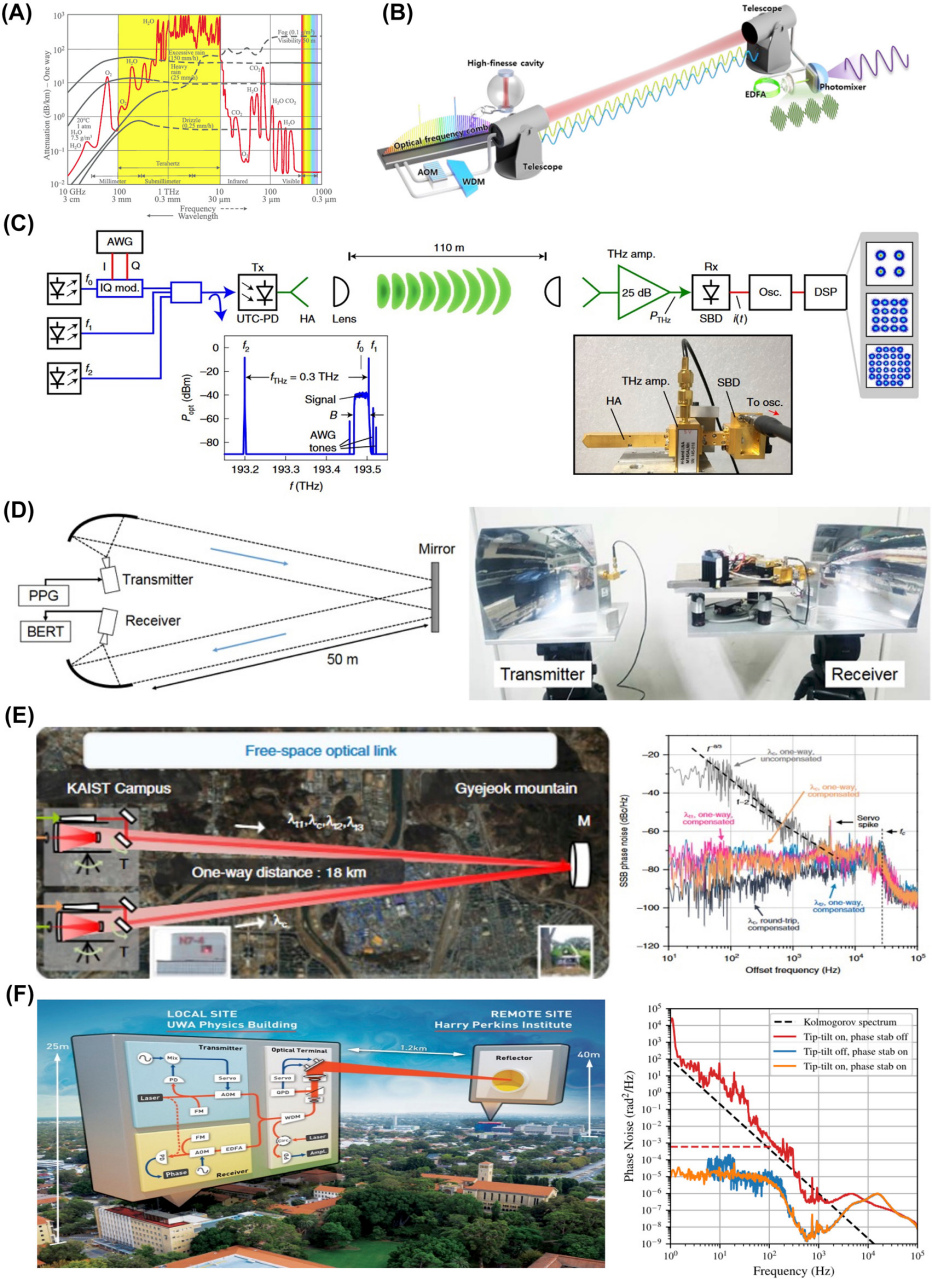
Examples of terahertz (THz) and free-space optical (FSO) links. (A) Atmospheric attenuation across the spectrum, including visible, terahertz, and radio-frequency regions [14]. (B) Achieving coherent transfer through a free space optical link and synthesizing an ultra-stable terahertz (THz) source. (C) Demonstration of coherent communication utilizing the Kramers–Kronig scheme over a 110 m THz link [37]. (D) Coherent communication through a 100 m distance link with a THz signal from filtered optical frequency comb modes [39]. (E) Coherent transfer of multiple comb-rooted optical frequencies over an 18 km link [125]. (F) Demonstration of active optics to suppress amplitude noise from the 2.4 km atmospheric link [127]. (A) Reproduced with permission [14]. Copyright 2011, De Gruyter. (C) Reproduced with permission [37]. Copyright 2020, Springer Nature. (D) Reproduced with permission [39]. Copyright 2016, IEEE. (E) Reproduced with permission [125]. Copyright 2019, Springer Nature. (F) Reproduced with permission [127]. Copyright 2022, American Physical Society.

In THz communication, the primary constraint lies in atmospheric attenuation [28]. The choice of frequency bands in the THz range is influenced by selecting regions with minimal atmospheric attenuation, directing attention toward the utilization of these specific bands (Figure 5A). The predominant factor contributing to the highest attenuation for THz radiation is atmospheric water molecules, and it is crucial to select bands with minimal attenuation, given the impact of humidity and weather conditions on propagation characteristics. This stands in contrast to the radiofrequency and optical frequency bands traditionally used for communication. Frequency bands in the range of 0.1–1 THz are under consideration, with key candidates being around ∼100–150 GHz, <350 GHz, and <500 GHz [28]. Considering attenuation, these bands are being explored for communication methods with respective transmission ranges of long-distance (∼1–10 km), intermediate-distance (∼0.1–1 km), and indoor communication (∼10–100 m). Due to atmospheric attenuation and free space path loss [28], antennas or amplifiers with high gain are essential, yet their development remains limited. While THz communication is gaining significance due to its effective communication speed enhancement (∼100 Gb/s–1 Tb/s) and potential for transmission over several kilometers, there are still limitations based on the frequency band, leading to varying communication ranges.

On the other hand, radio-frequency (RF) and optical frequency bands traditionally used for communication exhibit relatively low attenuation compared to THz, allowing for long-distance communication on the order of tens of kilometers. In the case of RF, however, challenges exist due to saturation in communication bands and lower carrier frequencies compared to THz and optical frequencies. Consequently, there is an expectation for the utilization of broadband high-speed communication using THz or optical frequencies. The THz range, positioned between RF and optical frequency bands, enables the generation of THz radiation through methods such as RF multiplier chain or optical frequency down-conversion. Compared to RF-based THz technology, photonic-based THz technology offers several advantages. Its broader bandwidth facilitates the transmission of large data volumes, and the stability achieved through frequency down-conversion ensures a robust signal with a superior signal-to-noise ratio (SNR). The use of a frequency comb for THz generation provides additional benefits, including traceability to time and frequency standards [41], concurrent multi-channel capabilities for enhanced wireless communication data rates [36], [65], and the ability to employ coherent optical links through fiber-optic [123], [124] and free-space optical (FSO) links [119], [125]. This effectively compensates for induced phase noise during transmission. Moreover, despite its complexity, the dissemination of optically clock-stabilized frequencies to remote sites becomes feasible, highlighting the versatility and potential of photonic-based THz technology in addressing the challenges posed by traditional communication frequencies.

Figure 5B illustrates a phase-coherent and extremely stable THz distribution technology through FSO links. This link, developed for optical communication, covers medium to long distances of 18 km [125] or 1.3 km [119], enabling phase-coherent transmission in the optical frequency range and stable THz generation at remote sites. This technology can be actively utilized in scenarios where transmission based on optical fiber links is not feasible and can ensure network flexibility, including terrestrial and nonterrestrial network configurations. Two optical frequencies, ν_1_ and ν_2_, are combined into a single beam through a single-mode optical fiber and launched to the remote site using a refractive optical transceiver with a 40 mm aperture diameter. Another identical refractive optical transceiver installed at the remote site focuses the beam into a single-mode optical fiber for reception. To counteract the power attenuation occurring during outdoor transmission between the transmitter and receiver, a pair of Er-doped fiber amplifiers (EDFAs) is employed at both Site A before transmission and Site B after reception to provide power boosting. To address phase noise encountered in atmospheric transmission, a portion of the optical frequency ν_1_ is sent back to Site A via the same FSO link in the reverse direction. The returned optical frequency is mixed with its original frequency to quantify atmospheric phase noise in terms of the Doppler RF beat. Assuming insignificant wavelength-dependent variations in the air refractive index in the 1550 nm near-infrared light band, another frequency, ν_2_, is expected to experience almost the same Doppler shift as ν_1_. Consequently, both frequencies can be monitored and controlled together, with an acousto-optic modulator (AOM) used to precompensate the Doppler RF beat of frequency ν_1_ through phase-locked loop (PLL) control. At the same time, at Site B, final THz waves will be synthesized by the transferred two optical frequencies ν_1_ and ν_2_ by heterodyning both frequencies.

THz communication networks can be broadly categorized into terrestrial and nonterrestrial networks, each serving specific applications. Terrestrial networks encompass both macroscale and nanoscale links, with applications within Earth’s atmosphere. Noteworthy applications of THz communications include front- and back-hauling of base stations in femtocells, wireless local area networks in smart offices, near-field communications like wireless connections in data centers, and device-to-device communications [28]. Nonterrestrial networks include ground/space links and space links, involving intersatellite and deep-space communication [126]. Challenges associated with THz space links include spreading loss due to electromagnetic wave propagation and high atmospheric attenuation in Earth-to-space links, limiting the utilization of the high THz band. However, for artificial satellites and space communication in less dense atmospheres, such as those on Mars, the low atmospheric attenuation creates opportunities for utilizing the THz band, offering high data rates.

Figure 5C shows a simplified coherent receiver approach for THz data signals [37]. This method involves utilizing an envelope detector and digital signal processing (DSP) for phase reconstruction based on the Kramers–Kronig (KK) receiver concept. In the experimental setup, a high-speed Schottky-barrier diode (SBD) functions as the envelope detector, showcasing the transmission of QPSK and 16QAM signals at a data rate of up to 132 Gb/s, with a carrier frequency of 0.3 THz. To achieve a long-distance wireless link of 110 m, horn antennas and 25 dB THz amplifiers were employed. Figure 5D demonstrates a real-time 300-GHz wireless link using a coherent transmitter and heterodyne receiver [39]. The experiment achieves a 100-m wireless transmission with a single-channel data rate of 50 Gb/s. The method involves filtering two modes from an optical frequency comb to generate a THz signal via photomixing and shows the potential of THz communication based on the optical frequency comb.

Figure 5E introduces a comb-rooted scheme designed to transfer multiple optical carriers concurrently across an 18 km open-air optical link [125]. The length of the optical link simulates turbulence effects observed in ground-to-satellite transmissions within the troposphere. The investigation showcases the simultaneous transfer of multiple comb-rooted optical carriers, facilitating long-distance delivery not only of individual optical frequencies but also of synthesized microwaves. This methodology capitalizes on high intercomb-mode coherence, presenting a versatile approach for efficient and robust data transmission over extended distances, especially in the presence of atmospheric turbulence. Figure 5F demonstrates successful phase- and amplitude-stabilized free-space frequency transfer over a 2.4 km atmospheric link, employing phase-locked loop (PLL) control and active optics [127]. Extrapolating from the results, it is anticipated that a ground-to-space link with atmospheric stabilization technology could achieve fractional frequency stability of approximately 1 × 10^−20^ within the viewing window of a low Earth orbit satellite transit. This advancement marks a significant step toward establishing a global network of optical atomic clocks for precise timescale comparisons in ground-to-space laser links through turbulent atmospheres.

## 6 Precision evaluation of 6G THz nano/microphotonic devices

**Figure 6:**
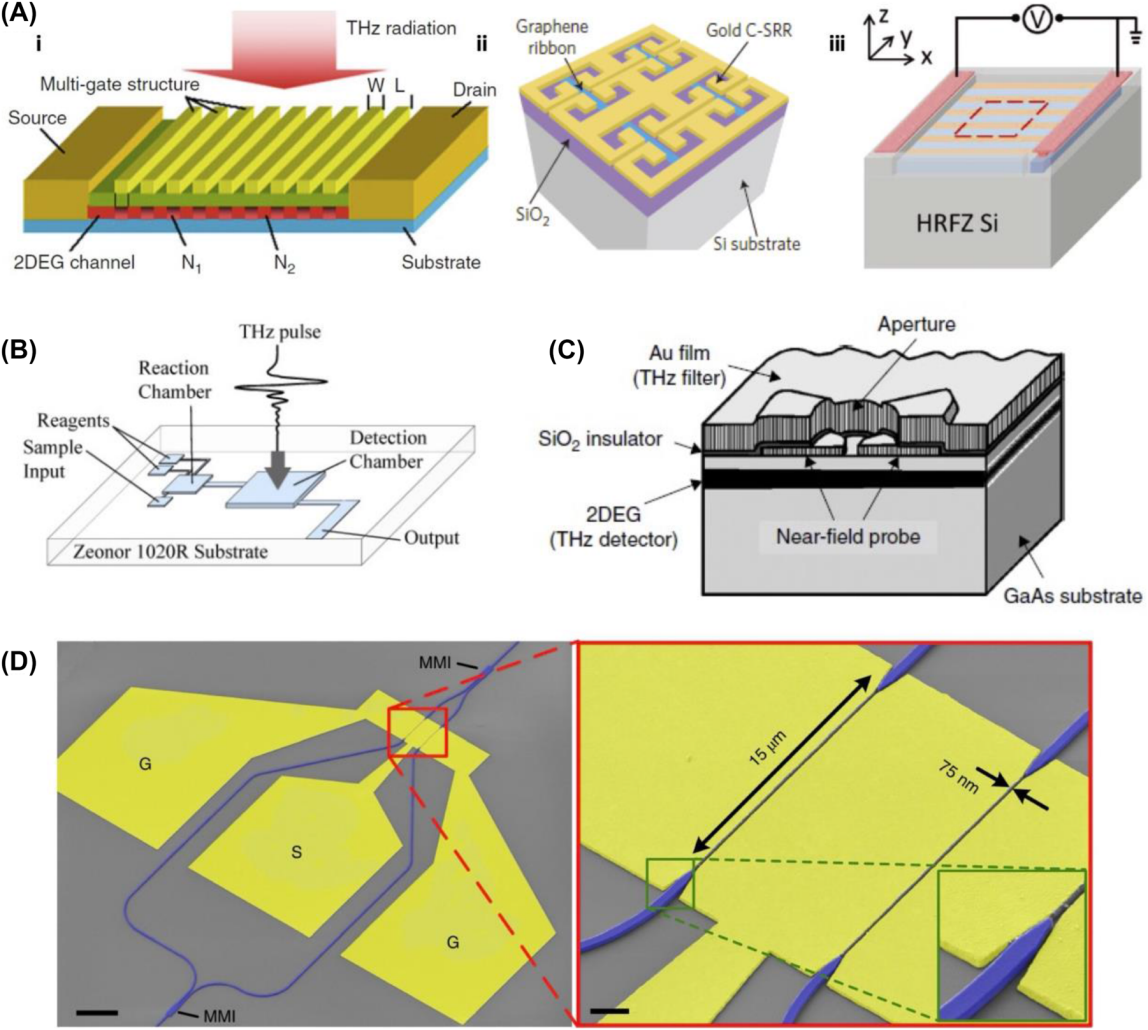
Schematic illustration of nano/micro devices for THz region. (A) Schematic of terahertz nano/micro applications; (i) multiple gates on a common channel [131], (ii) graphene-based metamaterial structured terahertz modulator [28], (iii) tunable silicon metasurface for broadband terahertz antireflection [132]. (B) Microfluidic devices for terahertz spectroscopy [130], (C) schematic representation of the integrated near-field THz imager [128]. (D) Plasmonic Mach–Zehnder modulator (MZM) with ground-signal-ground (GSG) contact pads (yellow) and silicon photonic waveguides (blue), scale bar, 20 μm [38]. (A-i) Reproduced with permission [131]. Copyright 2012, Springer Nature. (A-ii) Reproduced with permission [28]. Copyright 2016, Springer Nature. (A-iii) Reproduced with permission [132]. Copyright 2018, John Wiley and Sons. (B) Reproduced with permission [130]. Copyright 2008, Optica Publishing Group. (C) Reproduced with permission [128]. Copyright 2013, Elsevier. (D) Reproduced with permission [38]. Copyright 2019, Springer Nature.

The unique properties of THz sources have spurred extensive research into nano/micro application devices, employing intricately crafted nano/microstructures produced through advanced semiconductor processes (Figure 6A). These applications span diverse fields, including spectral sensing, imaging, and wireless communications. Distinguished by their lower frequencies compared to the conventional optical spectrum, THz waves are harnessed to detect hazardous substances [128]. Their selective transmission properties enable penetration through materials such as polymers, clothing, and wood, while rendering them opaque to metals, water, and glass. This capability has proven instrumental in security applications. The significance of THz frequencies extends to biosensing, as their spectral range aligns with the vibrational modes of substantial molecules like proteins and DNA [129]. In this context, micro/nanoscale devices have been designed for precise detection of DNA sequences. Notably, a microfluidic chip, depicted in Figure 6B, has been developed for THz spectroscopy, streamlining the detection of DNA information without the additional processing typically required for general DNA detection [130].

Image mapping technology, utilizing the analysis of received frequencies of THz waves reflected from materials, stands out as a nondestructive analysis method capable of determining the physical and chemical properties of materials. The exceptional transparency of THz wavelengths positions them as ideal light sources for near-field imaging [27]. Near-field imaging measurements beyond the diffraction limit by detecting the local extinction near-field behind an aperture [15]. This phenomenon occurs when electromagnetic waves are directed at an aperture smaller than the wavelength. Despite various designs for near-field measurement, ongoing research focuses on superimposing the THz evanescent field by integrating a micro/nano-sized THz detector at the rear of the aperture. Figure 6C illustrates a chip with a GaAs/AlGaAs heterogeneous structure and an 8 μm aperture. GaAs chips, featuring a two-dimensional electron gas (2DEG) layer at the heterointerface, allow for the direct measurement of the THz evanescent field using a 2DEG detector. This unique structure not only enhances sensitivity to detection but also presents a robust system, preoptically aligned due to the chip’s structural shape. The spatial resolution of the THz device is measured at 9 μm, significantly surpassing the diffraction limit of 107.3 μm at the incident wavelength of 214.6 μm. Moreover, variations in aperture size offer the capability for THz imaging with resolutions as fine as 500 nm [128], [133].

The success of future 6G wireless communication networks hinges on their ability to handle data rates ranging from tens to hundreds of Gb/s and operate at frequencies characterized by low atmospheric attenuation. Therefore, a crucial component for 6G commercialization is the establishment of a system utilizing carrier frequencies in the THz spectrum [28], [38]. This necessitates the integration of existing optical fiber–based infrastructure and processing methods for THz communication. A pivotal aspect of this integration is the connection between optical fiber and wireless THz communication devices [134]. Figure 6D illustrates the manufacture of a device designed for the direct O/T (optical to THz) and T/O (THz to optical) conversion of THz transmitters (Tx) and receivers (Rx). This device features a 500 nm Si nanowire waveguide and a 75 nm wide gold brush slot structure, created through a Si-based lithography process. Operating at a line rate of up to 50 Gb/s, this system transmits signals with a carrier frequency of 0.2885 THz, covering 16 m. The THz signal is generated through O/T conversion in the UTC-PDs. On the receiving end, the THz signal undergoes conversion to the optical domain using an ultra-wideband plasmonic–organic hybrid (POH) modulator. These POH modulators offer a flat frequency response of up to 0.36 THz, coupled with a compact footprint of approximately 600 μm^2^. This characteristic makes them well-suited for high-density photonic integration [38].

## 7 Summary and future prospects

The advent of next-generation 6G communication holds immense potential for transforming data transfer capabilities and enabling enriched sensory experiences in eXtended Reality (XR). This review emphasizes the critical role of advanced components operating in the terahertz (THz) regime, particularly within the frequency range of 0.1–1 THz, in achieving these advancements. However, existing evaluation tools for this frequency range exhibit limitations, prompting a focus on emerging trends and precision measurement techniques using optical frequency comb in the photonic regime. As 6G THz communication components grow in complexity, the establishment of THz frequency standards becomes imperative for accurate characterization. These standards play a pivotal role in evaluating the amplitude and phase characteristics, ensuring the development of reliable and high-performance 6G THz communication systems. Calibration techniques and measurement methodologies in the THz domain are emphasized for ensuring quality and interoperability.

6G communication is promising as we anticipate unprecedented connectivity and data throughput. Advanced components, such as waveguides, modulators, filters, amplifiers, and detectors, form the foundation of 6G networks, with innovations like metamaterials and low-loss dielectrics playing a crucial role. The miniaturization and integration capabilities of these components are pivotal for compatibility with compact and high-density 6G device designs. THz spectroscopy emerges as a potent tool for exploring various physical phenomena and finding applications in the analysis of THz devices. However, traditional THz spectroscopic methods, like THz time domain spectroscopy (THz-TDS), exhibit limitations. The future progress of THz applications relies on the development of high-efficiency interconnecting technology, emphasizing the need to meticulously evaluate the frequency and phase characteristics of these components.

Enabling ultra-stable THz signals is crucial for advancing 6G wireless communication. However, the challenge lies in achieving high-performance THz wave generation while adhering to international time standards, requiring sophisticated stabilization within a substantial system volume. Future prospects involve the phase-coherent distribution of these ultra-stable THz signals across extensive distances, forming the backbone of a robust 6G communication network. This can be achieved through diverse means, such as fiber networks or free-space optical links, each with its stability considerations and potential applications, including satellites, HAPS, and drones, underscoring the evolving landscape of 6G connectivity. The unique properties of THz sources have stimulated extensive research into nano/micro application devices, leveraging intricately crafted nano/microstructures produced through advanced semiconductor processes. This ongoing exploration highlights the potential for innovative applications and advancements in nano/microscale devices harnessing THz sources. Overall, the integration of advanced components, precise measurement techniques, and emerging technologies in the THz regime holds the key to accelerating the realization of high-density next-generation 6G communication.
